# Supplementary material for: LncRNA NEAT1/miR-146a-5p Axis Restores Normal Angiogenesis in Diabetic Foot Ulcers by Targeting mafG
Source: Cells. 2024 Mar 5;13(5):456. doi: 10.3390/cells13050456 (PMC10931324; doi:10.3390/cells13050456)
Supplement: Supplementary file 1 [file cells-13-00456-s001.zip › cells-2845237-Supplimentary information .pdf]

Epigenetic Regulation by the lncRNA NEAT1/miR-146a-5p Axis in Restoring Normal Angiogenesis through MafG Targeting in Diabetic Foot Ulcers

TCA Architha[1], George Raj Juanitaa[1], Ramanarayanan Vijayalalitha[1], Ravichandran Jayasuriya[1], Gopinathan Athira[2], R Balamurugan[2], Kunka Mohanram Ramkumar[1],*

[1]Department of Biotechnology, School of Bioengineering, SRM Institute of Science and Technology, Kattankulathur 603 203, Tamil Nadu, India.

[2]SRM Medical Hospital and Research Centre, SRM Institute of Science and Technology, Kattankulathur 603 203, Tamil Nadu, India.

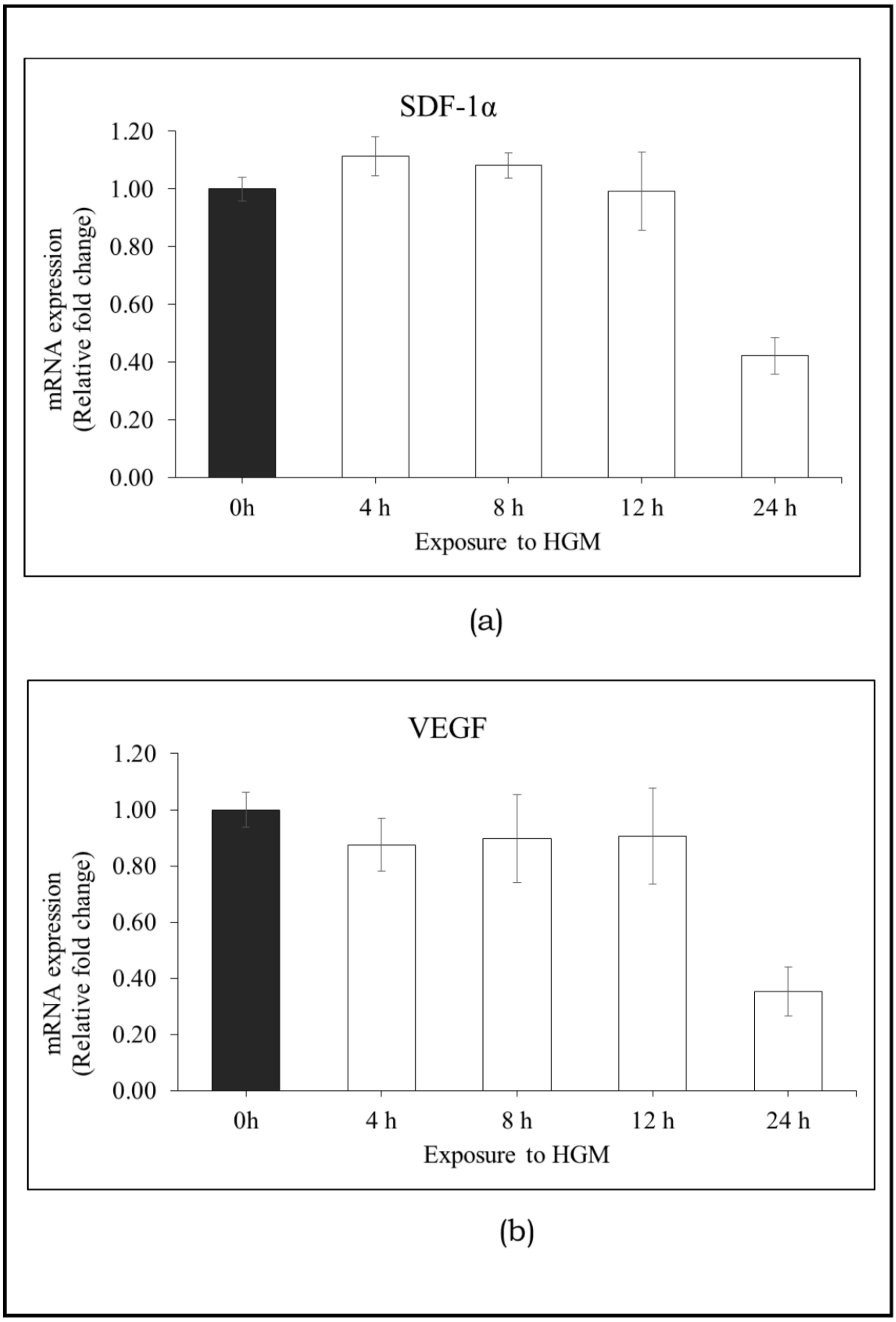

Figure S1: Expression of angiogenic markers such as SDF-1α (a) and VEGF (b) in HGM-induced endothelial cells at different time intervals.
